# The Role of Exo-miRNAs in Cancer: A Focus on Therapeutic and Diagnostic Applications

**DOI:** 10.3390/ijms20194687

**Published:** 2019-09-21

**Authors:** Francesco Ingenito, Giuseppina Roscigno, Alessandra Affinito, Silvia Nuzzo, Iolanda Scognamiglio, Cristina Quintavalle, Gerolama Condorelli

**Affiliations:** 1 Percuros BV, Albinusdreef 2 - 2333ZA Leiden, Percuros BV, Zernikedreef 8. 2333 CL Leiden, The Netherlands; francesco.ingenito@outlook.it (F.I.); a.affinito@percuros.com (A.A.); 2 Department of Molecular Medicine and Medical Biotechnology, “Federico II” University of Naples, Via Pansini 5, 80131 Naples, Italy; giusy_roscigno@yahoo.it (G.R.); Iolanda.scognamiglio@gmail.com (I.S.); 3 IRCCS SDN, IT-80143 Naples, Italy; snuzzo@sdn-napoli.it; 4 IRCCS Neuromed – Istituto Neurologico Mediterraneo Pozzilli, Via Atinense, 18 – IT-86077 Pozzilli (IS), Italy

**Keywords:** exosome, microRNA, tumor microenvironment, cancer therapy, cancer diagnosis, brain tumors

## Abstract

Exosomes are extracellular vesicles released into biological fluids where they act as carriers of various molecules, including proteins, lipids, and RNAs, between cells, modulating or perturbing specific physiological processes. Recently, it has been suggested that tumoral cells release excessive amounts of exosomes that, through their cargo, promote tumor progression, stimulating growth, angiogenesis, metastasis, insensitivity to chemotherapy, and immune evasion. Increasing evidence highlights exosomal microRNAs (exo-miRNAs) as important players in tumorigenesis. MicroRNA (miRNA) are a class of small non-coding RNA able to regulate gene expression, targeting multiple mRNAs and inducing translational repression and/or mRNA degradation. Exo-miRNAs are highly stable and easily detectable in biological fluids, and for these reasons, miRNAs are potential cancer biomarkers useful diagnostically and prognostically. Furthermore, since exosomes are natural delivery systems between cells, they can be appropriately modified to carry therapeutic miRNAs to specific recipient cells. Here we summarize the main functions of exo-miRNAs and their possible role for diagnostic and therapeutic applications.

## 1. Introduction 

Over the last decades it has been shown that cells, in addition to soluble mediators like hormones and cytokines, release more complex messages, including a particular type of secreted vesicles called extracellular vehicles (EVs) [1].

EVs are highly heterogeneous and therefore their characterization and classification has resulted tricky. According to their biogenesis it is possible to distinguish two big groups of EVs: exosomes and microvesicles. Exosomes are generated upon inward budding of the endosomal membrane with the plasma membrane; in contrast microvesicles are generated upon outward budding of the plasma membrane [2].

EVs carry functional molecules, such as membrane and cytosolic proteins, lipids, and RNA, and with their cargo contribute to metastatic niche formation and resistance to therapy [3]. 

In terms of quality and quantity, EV cargo is cell-type specific and changes according to the physiological or pathological state of the cell [4]. For this reason, EVs have been deeply investigated in body fluids such as serum, plasma, saliva, and liquor as novel biomarkers of human diseases. Once released into the extracellular space, EVs reach target cells and deliver their cargo, promoting phenotypic changes [5]. 

Since exosomes are a more homogeneous group among the EVs, they have been intensely investigated. 

MicroRNAs are a class of small, 17-24nt long noncoding RNA that induce post transcriptional gene silencing by binding the 3’UTR (untranslated region) of target genes. They are frequently dysregulated in cancer [6,7,8,9,10,11,12]. Extracellular microRNAs released by cells are packed in exosomes and microvesicles in order to gain greater stability and hinder their degradation. 

Since exosomes are potential diagnostic biomarkers, characterization of microRNAs transported and transferred within exosomes could highlight new mechanisms in cancer progression and lead to the identification of new biomarkers. In this review, we discuss the functional role of microRNAs on tumor progression and microenvironment equilibrium, focusing our attention on exosomes as vectors for intercellular delivery of microRNAs.

## 2. Exosomes: General Features

Exosomes are endosome-derived extracellular vesicles with a diameter between 30–100 nm [13] that act as carriers of different biological materials, such as proteins, lipids, and nucleic acids between cells located also in distant regions of the body.

Exosomes originate from the maturation of endosomes within the cytosol, and part of the endocytic cargo consisting of extracellular and cellular components can be incorporated into small vesicles born by inward budding of early endosomes, generating a new type of cytoplasmic vesicle known as a multivesicular body (MVB) [3]. 

**Figure 1:**
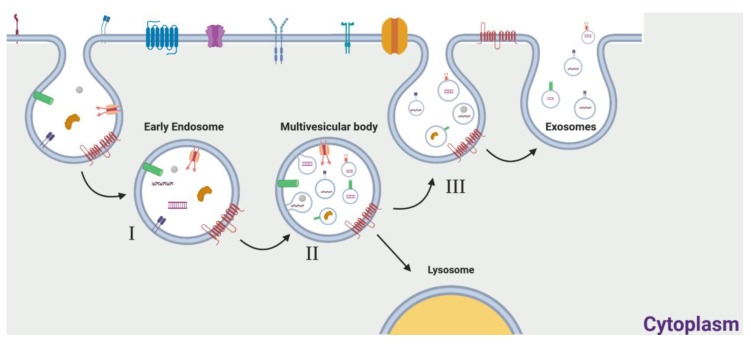
Schematic representation of exosome formation and release. Exosomal cargo from the extracellular and intracellular spaces is transported to early endosomes entering the endocytic pathway (I). During endosome maturation, the early endosomal membrane invaginates, leading to the formation of a late endosome, known as a multivesicular body (MVB), which is characterized by the presence of small, internal microvesicles. By this process, cargo is internalized into these microvesicles, now called exosomes (II). MVBs preferentially fuse with lysosomes, for the degradation of cellular components (III), or can fuse with the cytoplasmic membrane, allowing the release of the exosomes into the extracellular space (IV).

Since MVB formation requires internal invagination of the endosomal membrane, membrane proteins retain the same orientation they have on the plasmatic membrane, enclosing the inner endosomal components into the small vesicles [14]. The fate of MVBs is fusion either with lysosomes, for degradation processes, or with plasma membrane (PM), which causes the release into the extracellular space of these inner vesicles, known as exosomes (Figure 1) [3,15].

The selective sorting of distinctive proteins and nucleic acids into exosomes is mediated by a variety of mechanisms, most of which are not fully characterized. Concerning proteins, their specific sorting is controlled by mechanisms that involve ESCRT, tetraspanins, and lipid-dependent machineries [16]. Much evidence shows that RNA sorting is mediated by proteins that recognize specific sequence regions, like in the case of hnRNPA2B1, which recognizes specific cis-acting elements of miRNAs, controlling sorting in a very precise way [17].

### The Role of Exosomes in Cancer

Cancer cells secrete a higher quantity of exosomes compared to healthy cells, and “tumoral exosomes” may transfer factors involved in cancer progression, perturbing the activity of receiver cells and favoring tumorigenesis through regulation of proliferation, escape from immune control, drug resistance, and metastatic processes [18]. Riches et al. described that, during 24 hours of incubation, normal breast epithelial cells release 4.5 ± 2.3 × 10^8^ exosomes per 10^6^ cells, while a breast cancer cell line produced 53.2 ± 1.6 × 10^8^ exosomes per 10^6^ cells during the same period [19]. Similarly, a study conducted by W. Zhang et al. reported that healthy ovarian cells were characterized by a sharp increase in exosome release for only up to 10 hours of incubation, while the tumoral cells taken into consideration produced exosomes for 10–16 hours [20]. 

Excessive release of exosomes can be induced not only by intrinsic cell properties, like mutations of genes involved in exosome biogenesis, but also by microenvironmental conditions.

H.W. King showed that hypoxia led to the induction of exosomal release in breast cancer cells. The exposure of these cells to modest (1% O_2_) and severe (0.1% O_2_) hypoxia led to 32.3  ±  4.8% and 90.9  ±  7.1% increases of exosomes into the media as compared to healthy cells [21]. A study on melanoma cells proved that exosomal release and uptake are improved in an acidic microenvironment: In particular, the rigidity and sphingomyelin/ganglioside GM3 content of exosomal membrane was higher at low pH, which involved increased fusion efficiency [22].

The importance of exosomal release and transfer have been validated in different types of cancer; in particular, they are emerging as key players in glioblastoma (GBM) genesis and progression. Exosomes in the central nervous system (CNS), which move factors between myelinating glia and neurons, are involved in processes such as neuronal survival, synapse assembly, and plasticity, [23].

However, GBM cells release exosomes that differ from those released in healthy conditions. An analysis conducted by J. Skog et al. identified various proteins such as angiogenin, FGF, IL-6, IL-8, TIMP-1, VEGF, and TIMP-2 involved in GBM angiogenesis and able to increase malignancy. Most of these factors presumably are released into the extracellular microenvironment (ECM) upon the lysis of the exosomes due to the acidic tumor environment and interacted with cognate receptors on the surface of endothelial cells to promote angiogenesis [24]. Furthermore, under hypoxic conditions, GBM cells release exosomes rich in VEGF-A, which increases permeability of the blood-brain barrier (BBB), reduces the expression of claudin-5, and facilitates angiogenesis [25].

Another example of an oncogenic factor loaded in exosomes is EGFRvIII, which has been detected in serum microvesicles from GBM patients [26]. The uptake of EGFR-expressing exosomes leads to the integration of the receptor to the plasma membrane of receiver cells and to subsequent activation of oncogenic signaling pathways, such as the ERK1–ERK2 and AKT pathways [27].

Tumoural exosomes promote cancer development and escape from immunologic control [28].

A study by Domenis R. et al. suggested that GSC-derived exosomes can inhibit activation and proliferation of T cells and production of Th1 cytokines, without perturbing their suppressive ability, by acting on monocytes by promoting a Mo-MDSC (monocytic myeloid-derived suppressor cell) tumor-supportive phenotype. Indeed, they demonstrated that GSC-derived exosomes are internalized by CD14+ monocytes, inducing the release of IL-1β, IL-6, and IL-10 [29].

Qu et al. showed that gastric cancer derived-exosomes facilitate metastasis by disrupting the mesothelial barrier through apoptosis and mesothelial-to-mesenchymal transition (MMT) of peritoneal mesothelial cells [30]. Furthermore, Hu et al. indicated that exosomal miRNAs from malignant ascites induce an invasive effect on gastric cancer cells; in particular, when ascites exosomes are released into the peritoneal cavity and internalized by circulating gastric cancer cells, they provoke an abnormal activation of the EMT signaling pathway, enabling peritoneal dissemination [31]. Another study by Qiu et al. underlined the role of exosomal long non-coding RNAs (lncRNAs) in metastasis progression. In particular, they demonstrated that metastatic epithelial ovarian cancer cells transfer the lncRNA MALAT1 via exosomes to umbilical vein endothelial cells, stimulating angiogenesis-related gene expression and empowering metastasis formation [32].

New evidence suggests that exosomes can alter the chemosensitivity of cancer cells by manipulating cellular signaling pathways. Liu et al. determined that doxorubicin resistance can be transferred from gastric-resistant cancer cells to non-resistant cancer cells by the horizontal transfer of exomiR-501, which seemed able to downregulate the cell-death inducer BLID (BH3-like motif-containing protein) [33]. Likewise, Fen et al. indicated that pancreatic tumor exosomes can transmit gemcitabine chemoresistance to sensitive cells within the same tumor or at other anatomical locations. This study proposes that the presence of EphA2 on tumoral exosomes could be responsible for this ability, revealing a previously unknown property of this protein [34].

Other than the presence of oncogenic proteins in tumoral exosomes, nucleic acids can take part in carcinogenesis. In particular, there is increasing evidence for miRNAs playing a role not only in cancer cell growth, but also in creating a favorable microenvironment for tumors development.

## 3. MiRNA and Exosomes

### 3.1. Extracellular miRNAs 

The human genome is made up of coding and non-coding regions. Some non-coding DNA is transcribed into regulatory RNA molecules, the best studied of which are long non-coding RNAs (lncRNAs), which are over 200 nucleotide long, and microRNAs, which are short sequences (20–25 nucleotides) known to regulate 50% of protein-coding genes. MicroRNAs regulate gene expression post transcriptionally by binding to the 3′ UTR of target genes and inducing translation repression or RNA degradation.

MicroRNAs are known to be deregulated in cancer, and several microRNAs are reported to act as oncogenes or tumor suppressors. For example, miR-221 and miR-21 are well-documented oncomiRNAs that exert anti-apoptotic effects and induce cell proliferation and an aggressive phenotype [35]. 

On the other hand, the miR-34a and let7 families inhibit tumor growth and stimulate apoptosis [36,37]. Other miRNAs are able to confer resistance to chemotherapy, as in the case of miR-24, which increases the resistance to cisplatin of breast cancer stem cell by inhibition of its pro-apoptotic target Bim L [11], and miR-221/222, overexpressed in high-grade GBM patients, not only inhibit the expression of the tyrosine phosphatase PTPμ, regulating positively cancer cell migration [9], but also increase temozolomide resistance through modulation of expression of the DNA repair enzyme MGMT [10].

While the majority of miRNAs are found in the intra-cellular compartment, recently a high number of miRNAs (known as circulating miRNAs) have also been detected in the extracellular compartment: They have been found in body fluids, such as serum [38,39] or plasma [40,41], saliva, urine, breast milk, cerebrospinal fluid, and seminal fluid [42]. Surprisingly, these circulating miRNAs are remarkably stable and can survive under unfavorable conditions for long time. Interestingly, the stability of endogenous miRNAs contrasts with that of synthetic spike-in miRNAs, which are rapidly degraded in plasma.

MiRNAs are protected from RNase activity by different mechanisms: wrapped in membrane vesicles (exosomes, microparticles, and apoptotic bodies), associated with proteins, or enclosed in lipoprotein complexes [43,44]. The first authors reporting on exosomes carrying miRs were Valadi et al. In their pioneering work, they found that exosomes contain heterogeneous RNA species, including miRNAs. Furthermore, they discovered that exosomes can contain higher concentrations of miRNAs, such as let-7, miR-1, miR-181, and miR-375, compared to the cells of origin, indicative of selective wrapping of miRNAs in the exosomes. [45]. Then, in 2008 Hunter et al detected microRNAs in peripheral blood vesicles. These two discoveries opened up the path for a revolutionary theory: microRNAs can be loaded and transported to distant sites through exosomes [46]. Since then, in addition to their role in intracellular signaling pathways, microRNAs have been identified as key mediators of cell-cell communication, playing crucial roles as autocrine, paracrine, and endocrine signaling regulators. 

### 3.2. Passive and Selective Release of miRNAs from Cells to Exosomes

**Figure 2:**
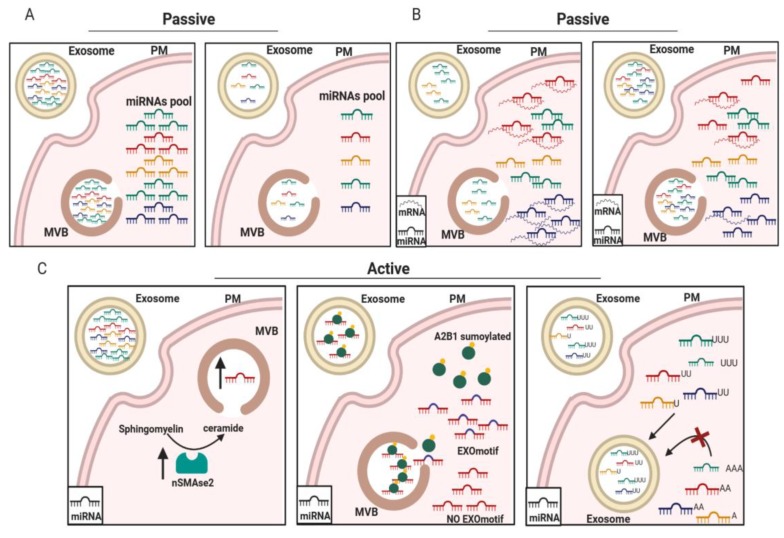
Active and passive mechanisms of miRNA loading in exosomes. miRNAs are more or less present in exosomes on the basis of either their relative abundance in cells (**A**) or the abundance of their target mRNAs (**B**). (**C**) Active mechanisms of miRNA loading are regulated by nSMAse2 (left panel) or by sumoylated A2B1, which recognizes an EXO motif present in the miRNA sequence (middle panel), or by a specific sequence containing uridine at the 3’UTR of the miRNA (right panel).

Several studies have demonstrated that microRNAs can be packaged into exosomes via two mechanisms. The first is a passive mechanism dependent upon the cellular microRNA expression profile: the microRNAs are packaged passively, according to the amount present in the cell of origin (Figure 2A,B). Pigati et al. [47] were the first to focus investigation on the mechanism by which miRNAs are packed into exosomes. The authors performed a microarray analysis comparing intracellular and exosomal microRNAs in MCF7 cells. They found that about 66% of the miRNA level present in the exosomes was proportional to the intracellular microRNA content (Figure 2A). For example, the amount of miR-638 in exosomes closely reflected its level within the cell. Moreover, also the transcriptomic changes that occur in cancer or in activated cells may influence the amount of the miRNAs loaded into exosomes. The presence, for example, of a high number of miRNA target transcripts, could differentially modulate the amount of miRNA engaged by gene silencing or by MVB biogenesis (Figure 2B) [48].

Pigati and colleagues also observed that some miRNAs, such as miR-451 and miR-1246, produced by cancer mammary cells are released independently from their intracellular levels, whereas the majority of the described miRNAs produced by non-malignant cells are retained in the cells. This work opened the idea that, in addition to the passive release mechanism based on the miRNA cells amount, cells have a selective miRNA-release mechanism that is cell-dependent [47]. 

In this regard, there are at least three potential active mechanisms for the sorting of miRNAs into exosomes. The first mechanism involves a neutral sphingomyelinase 2 (nSMase2)-dependent pathway. nSMase2 is involved in the synthesis of ceramide, a phospholipid enriched in exosomal membranes and required to promote exosomal biogenesis. Kosaka et al. found that the inhibition of nSMAse2 inhibits not only the formation of exosomes but also the amount of miRNAs uploaded (Figure 2C, left panel) [49,50,51].

The second mechanism was elucidated by Villarroya-Beltri et al. The group identified short sequence motifs (GGAG, UGAG, CCCU, and UCCU) present in the 3′ end region of the miRNAs sequences that guide their packaging into exosomes in human primary T lymphocytes. Furthermore, they found that the sumoylated chaperone protein named hnRNPA2B1 bound miRNAs presenting with “EXOmotifs” and promoted the loading of microRNAs into exosomes (Figure 2C, middle panel) [17]. The third mechanism is related to post-transcriptional modifications of the miRNAs at their 3’ end. It was found that exosomes secreted by B-cells or isolated from human urine were enriched in miRNAs with uridylated 3’-ends, while those containing adenylated 3’-ends were overrepresented in the cells of origin (Figure 2C, right panel) [52]. 

In addition to physiological events, cancer can alter microRNA packaging within exosomes. Although a specific mechanism linking changes in microRNA content in exosomes has not yet been identified, it is clear that cancer alters signaling pathways that control miRNA loading in exosomes. For instance, in colorectal cancer cells, mutation of KRAS has been shown to influence exosomal cargo through an nSMAse-dependent mechanism, with mutant KRAS cells loading preferentially a greater amount of miR-100, whereas wild-type KRAS cells package higher levels of miR-10 in the exosomes [53].

### 3.3. Exosomal miRNAs Involvement in Cancer

**Figure 3:**
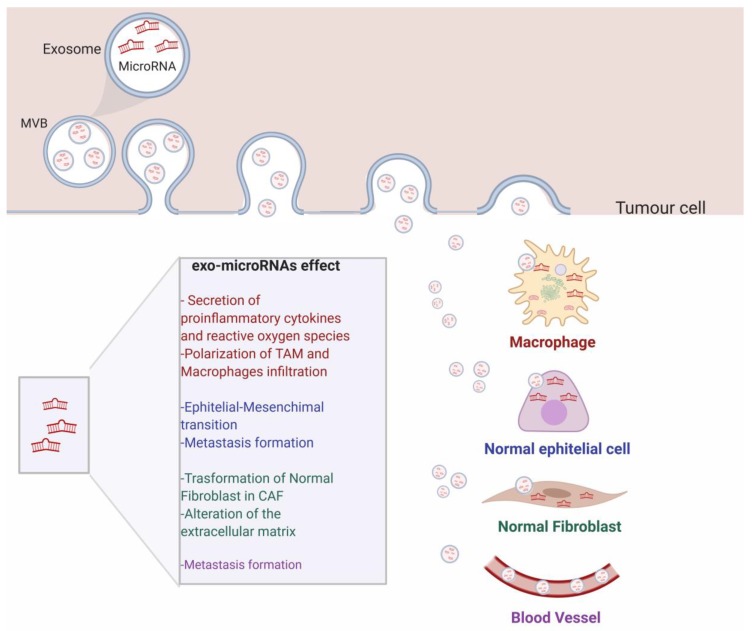
Schematic representation of the roles of exo-miRNAs on distant cells and in the cancer microenvironment. Exo-miRNAs released by tumor cells move to the extracellular space to reach near-by cells, such as macrophages, normal epithelial cells, and normal fibroblast. The exosomal cargo is responsible for cellular transformation and enhanced cancer progression**.** Exosomes can also reach distant cells, by moving along the blood stream.

Recent evidence suggests that exosomes are involved in the transferring of miRNAs from donor cells to adjacent cells, acting as messengers between tumoral and stromal cells [54]. This reciprocal communication can induce the reprogramming of the target cells’ gene expression, altering tumor growth, metastasis, epithelial-mesenchymal transition [55], angiogenesis, and immune function [56] (Figure 3). For instance, a recent work by Zhang et al. demonstrated the existence of reciprocal cross-talk between tumor and the microenvironment through exo-miRNAs that support tumor proliferation and apoptosis inhibition thanks to induced loss of PTEN [57]. Another example is represented by miR-660-5p. This miR is released by exosomes and promotes tumor proliferation and viability by targeting KLF9 and, thus, sustains progression of Non-Small Cell Lung Cancer (NSCLC) [58]. Moreover, studies have proved not only that different exo-miRNAs can regulate cancer aggressiveness and progression, but a single miRNA can impact different cancerous aspects at the same time. For instance, in breast cancer, miR-155 induces exosome-mediated chemoresistance to doxorubicin and paclitaxel, and simultaneously act as an oncogenic signal reprogramming cancer metabolism [59,60].

Recently, it has been shown that exosomal microRNAs released from cancer cells can influence angiogenesis by effecting cells of the tumor microenvironment or the mesenchymal stem cell population. Paggetti et al. showed that chronic lymphocytic leukaemia cells released exosomes enriched in miR-21, miR-155, miR-146a, miR-148a, and let-7g, which are taken up by endothelial and mesenchymal stem cells. The transfer of the exosomal cargo induces transcriptomic changes that promote an inflammatory phenotype in the stroma cells, which resembles the phenotype of CAFs (Cancer-associated fibroblasts) [61]. In addition, lung cancer cells have been shown to vehicle miR-21 via exosomes to surrounding normal epithelial cells. This led to an increase of VEGF levels, inducing angiogenesis. Interestingly, the inhibition of exosomal miR-21 decreased VEGF production in recipient cells, blocking exosome-mediated angiogenesis [62]. In a similar manner, during hypoxia, A549 lung cancer cells release miR-494-containing exosomes to nearby endothelial cells, enhancing angiogenesis through the suppression of PTEN and the Akt/eNOS pathway [63]. 

Metastasis is a complex process that involves different steps such as intravasion, migration in vascular system, extravasion and invasion of distant organs. Several studies illustrate that exosomal microRNAs released by cancer cells are able to enhance tumor cell migration and promote metastasis by inducing tumor cells plasticity and manipulating local stroma cells. The components of the mir-200 family (miR-200a, -200b, -200c, -141 and -429,) were classically considered as tumor suppressor genes, thanks to the ability to downregulate Zeb1 and block EMT [64]. Interestingly, Le MT et al. demonstrated that extracellular vesicles containing miR-200 can be transferred from metastatic to non-metastatic cells. MiR-200 promoted metastatic capacity to weakly metastatic cells and conferred the ability to colonize distant tissues, inducing EMT [65]. Another example is reported by Zhou et al [66]. The authors demonstrated that miR-105 is secreted by metastatic breast cancer and destroys the vascular endothelial barrier by targeting the cellular tight junction protein ZO-1 in endothelial cells, favoring premetastatic niche formation.

Recently, it is well established that tumor growth and progression are closely linked to the immune system. Cancer is able to reprogram immune cells (macrophages, dendritic cells, natural killer cells, and T cells) and suppress anti-tumor responses, promoting tumor survival, invasion, and progression. Secretion of exosomal miRs can play a crucial role in immune cell modulation, altering immune response. Tumor-Associated Macrophages (TAMs) are the predominant leukocytes infiltrating tumors and are known to produce cytokines that stimulate angiogenesis and the release of metalloproteases needed to degrade the extracellular matrix. It has been demonstrated by Fabbri et al. that miR-21 and miR-29a secreted by tumor cell exosomes can bind to TLR8 receptors expressed on macrophages, leading to NF-κB activation and secretion of inflammatory cytokines [67]. In contrast, TAMs can shuttle exosomes containing miR-223 to breast cancer cells, which promotes an aggressive and invasive phenotype of breast cancer cells. Other immune cells can also be target by exosomal communication. For instance, DCs (Dendrytic cells) are antigen-presenting cells that induce the activation of T-cells. Recently, it has been reported that pancreatic cancer cells inhibit DC activation by secreting exosomes enriched in miR-203. This miR is able to repress TLR4 expression and decrease the production of TNF-α and IL-12 [68]. Zhou M. et al. reported that nasopharyngeal carcinoma cells induce a suppressive tumor microenvironment, promoting regulatory T-cells (Treg cells) by releasing exosomal microRNAs (miR-24-3p, miR-891a, miR-106a-5p, miR-20a-5p, and miR-1908) [69]. Interestingly, it has been reported that Treg cells transferred miRNAs to T helper Th1 cells, suppressing Th1 cell proliferation and cytokine secretion through Ptgs2 inhibition [70]. Furthermore, miR-92b transferred through exosomes to natural killer (NK) cells suppresses CD69 expression and NK-mediated cytotoxicity by facilitating recurrence in hepatocellular carcinoma [71].

Additional oncosuppressive and tumor-promoting miRNAs present in exosomes are summarized in Table 1.

Taken together, these studies highlight that exosomal microRNAs secreted by tumoral cells can induce gene expression changes in recipient cells and promote tumor growth and metastasis by altering tumor cell plasticity and manipulating the tumor microenvironment.

## 4. Exosomal miRNAs as Biomarkers in Diagnosis and Prognosis in Cancer

Liquid biopsy is a pioneering, non-invasive diagnostic approach with the potential to identify new disease-associated biomarkers for earlier diagnosis.

Already in 1965, Gold et. al. demonstrated the possibility of using blood to identify cancer cells [85]. Since then, many serum cancer biomarkers have been identified, such as CA-125 in ovarian cancer and PSA (Prostate Specific Antigen) for prostate cancer. However, their diagnostic use is limited by low sensitivity [86]. Recently, researchers have isolated tumor-derived components from body fluids that could be used for diagnostic purposes, such as circulating tumor cells (CTCs), macrovesicles, and exosomes. 

The use of exosomes in liquid biopsy is becoming appreciated for its potential to predict different diseases, such as cancer. Here, we will discuss the diagnostic value and the state of art of exo-miRNAs in cancer.

Exo-miRNAs may represent interesting biomarkers in cancer because: (i) They are loaded into exosomes at higher levels in tumor cells [87]; (ii) they are stable at different temperatures [88]; and (iii) within exosomes [89], they are protected from RNase.

In biological samples, such as liquid biopsies, exo-miRNAs can be analyzed through different techniques. Quantitative real-time PCR is the gold standard to detect specific miRNAs. This method generates fluorescence signals proportional to the quantity of the PCR product and allow the detection of a small number of miRNAs. Microarray-based technologies, often used to check a large number of miRNAs, relies on hybridization of exosomal RNA to specific probes. More recently, next-generation sequencing (NGS) has become increasingly important for the detection of new miRNAs. The approach consists in RNA amplification followed by sequencing of the PCR products. The output data allows the identification of known and unknown miRNAs [90,91,92].

Exo-miRNAs can be considered as biomarkers for early detection and prognosis of cancer. For example, in 2017, Jin et al. selected four exosome-derived miRNAs (let-7b-5p, let-7e-5p, miR-23a-3p, and miR-486-5p) that were promising for early diagnosis of NSCLC. The profile of all identified microRNAs showed a sensitivity and specificity higher than 80% [93] (ref 80%) in NSCLC identification. In GMB, one important study by Manterola et al. described that the combination of two exo-miRNAs (miR-320 and miR574-3p) and RNU6-1 may discriminate GMB patients from healthy individuals [62]. In prostate cancer, miR-182 and miR-183 are highly expressed in cancer exosomes [94], while miR-1290 and miR-375 may predict prognosis in patients [95] with prostate cancer [96]. However, due to the simultaneous expression of some microRNAs in different types of cancer, their specificity is challenging. For example, exo-miR-21 is increased in breast cancer [97], in glioma [95], colorectal cancer [98], oesophageal squamous cell cancer [99], and ovarian cancer [100]. This suggests that some microRNAs, such as exo- miR-21, are not specific to one type of cancer, but could be considered as biomarkers for the presence or absence of cancer in general.

Moreover, the analysis of the exo-miRNA signature could be helpful in identifying cancer staging. For example, Kanaoka et al. used a retrospective analysis to identify exo-miR-451a as a promising biomarker to predict the prognosis in NSCLC patients at stages I, II, and III [101]. In breast cancer, exo-miR-223-3p is associated with histological type pT and pN, pathological stages of lymphatic invasion, and the nuclear grade (NG) of breast cancer, resulting significantly higher in the early stage [102]. This study was conducted on a small subset of 185 clinical biopsy samples; however, a large number of cases are needful to confirm the role of exo- miR-223-3p as a biomarker for BC detection. exo-miR-301a is considered a serum biomarker for diagnosis and recurrence of GMB. It correlates with the pathological grades and it is downregulated after surgical resection of the tumors, but its expression increases again at recurrence of tumor mass [103]. In ovarian cancer, Meng X et al. found higher levels of exosomal miR-200b and miR-200c in patients with FIGO (International Federation of Gynecology and Obstretics) stage III–IV compared to patients with FIGO stages I–II [104].

Other microRNAs have also been selected for their potential use as diagnostic serum biomarkers useful in discriminating different cancer subtypes. For example, exo-miR-373 was found more expressed in Triple-negative Breast Cancer (TNBC) than in the luminal subtypes, indicating that it may represent a novel specific biomarker of TNBC [105]. In NSCLC, Jin et al. identified four exo-miRNAs (miR-181-5p, miR-30a-3p, miR-30e-3p, and miR-361-5p) and three exo-miRNAs (miR-10b-5p, miR-15b-5p, and miR-320b) that may represent a specific signature of, respectively, adenocarcinoma and squamous-cell carcinoma [59].

Finally, some exo-miRNAs have been identified as recurrence-specific biomarkers. In particular, in NSCLC patients, miR-4257 and miR-21 are more expressed in patients undergoing recurrence after surgery compared to patients without recurrence [106]. 

In the last years, three clinical trials have been set up to investigate the role of the exosome content, including that of exo-miRNAs. In 2018, Lei Li started a prospective enrolment of high-grade serous ovarian cancer (HGSOC) and benign gynaecologic disease patients to identify, with the aid of next-generation sequencing (NGS), miRNAs and ncRNAs that were biomarkers for the detection and prediction of progression-free survival of ovarian cancer (ClinicalTrials.gov Identifier: NCT03738319).

In prostate cancer, the role of “prostasomes”, the prostate exosome cargo, is currently being investigated as a diagnostic tool in two clinical trials. Both of them aims to recruit patients with prostate cancer and analyze the prostasome content by NGS (ClinicalTrials.gov Identifier: NCT03694483, NCT03911999).

## 5. Exosomal miRNA-Based Therapy

Thanks to the particular features of exosomes—such as their ability to carry functional molecules over long distance within the body through the systemic circulation, their stability in the circulation, their compatibility with biological systems, and their ability to be recognized and taken up by specific recipient cells—they have recently been considered also for possible application in therapy. In fact, exosomes can be used as carriers for the transport of functional therapeutic molecules to unhealthy cells.

Over the last few years, different methods of exosome engineering for the encapsulation of therapeutic agents, like drugs or nucleic acids, have been developed. In particular, considering the abundantly demonstrated role of miRNAs in cancer regulation, exosomes have been identified as a valid method for the delivery of these nucleic acids to tumoral cells, opening the possibility of novel therapeutic approaches for cancer treatment. 

Schmittgen et al showed the therapeutic potential of exo-miRNAs on immune escape in neuroblastoma. The author demonstrated that NK cell-derived exosomes or nanoparticles can be used to deliver and restore miR-186 levels and, thus, reduce tumor size, restoring NK-mediated cytotoxicity [107]. As proved by Le’s group, exosomes can be engineered to deliver antisense oligonucleotides, usable as RNA drugs, to inhibit cell proliferation in leukaemia and breast cancer [108].

Moreover, given the crucial role that exosomes have in cell-cell communication and, thus, in cancer progression, new strategies have been drawn up aimed to inhibit exosome releasing or their uptake. The blockage of exo-miRNA cargo could be the result of rab27 silencing: rab27 depletion leads to a reduction of miR-494 abundance and, hence, to decreased tumor growth and metastasis in melanoma [109]. Another way to inhibit exosome release is obtained by treating cells with GW4869, a blocker of exosomes biogenesis [33,110]. Alternatively, the effects of exosomes can be reduced by inhibiting their uptake. In this regard, treatment with endocytosis inhibitors interrupt multiple myeloma cell communication with bone marrow stroma, reducing proliferation and drug resistance [111].

As already mentioned before, some registered clinical trials (clinicaltrials.gov database) on exo-miRNAs as diagnostic tools are currently ongoing. Additionally, a large number of trials have been registered to study the role of miRNAs or exosomes in patient outcome and therapy. Unfortunately, the field of therapeutic exo-miRNAs is at an embryonic stage, and no trials are available at the moment. 

However, an ongoing interventional study aims to recruit malignant glioma patients to conduct a phase I human trial (NCT02507583) using exosomes as the carrier of an antisense RNA oligonucleotide. The purpose of this study is to activate the immune system through exosomes released by glioblastoma cells treated with an Insulin-like growth factor receptor-1 Antisense Oligodeoxynucleotide and diffused by 20 diffusion chambers implanted in the rectum of the patients. Investigators believe that the resulting immune system activation can potentially have higher benefits and lower risks compared to current glioma treatment options. Another very recent study in metastatic pancreatic cancer patients uses mesenchymal stromal cell-derived exosomes delivering a small interference RNA (siRNA) against a mutated KRAS (NCT03608631). This study aimed to prove the clinical applicability of exosomes for metastatic pancreatic ductal adenocarcinoma treatment. Anyway, the studies mentioned above and many others highlight roles for exo-miRNA cargo in all the major aspects of cancer, such as tumor growth and progression. Above all, the important role in cancer progression of exosomal miRNAs make them highly interesting in the clinical scenario as they may provide the basis for novel strategies in cancer treatment. Therefore, the great interest on exosomal microRNAs expressed over the last years could pave the way to future clinical trials that will continue to implement clinical management of cancer patients.

## 6. Conclusions

Exo-miRNAs engender important mechanisms in the context of cellular communication, regulating physiological as well as pathological processes. Furthermore, there is increasing evidence of how tumoral cells use exosomes to deliver oncomiRNAs, miRNAs that promote cancer growth and spread within the body. Deeper knowledge of these miRNAs may lead not only to better comprehension of tumor progression, but also to new therapeutic approaches. The capacity to find new methods for more precise diagnosis and therapy of cancer is one of most important aims of modern medicine. Generally, current methods for tumor diagnosis fail in the identification of cancer at early stages, preventing timely treatment. Furthermore, the gold-standards of cancer therapies, chemotherapy and radiotherapy, present various problems, such as an inability to act specifically on cancer cells, causing various collateral effects. However, new progress in the field of diagnosis and precision medicine has been made over the last few years. The use of blood components for early diagnosis is gaining more importance. In particular, exo-miRNAs are emerging as promising biomarkers and valuable therapeutic agents. Indeed, exosomes and their miRNA cargos, on account of their being easily detected in biological fluids like blood, can provide a non-invasive way to detect the presence of tumoral cells, classify cancers, and forecast response to specific drug treatments, avoiding invasive diagnostic techniques like tissue biopsy. Improved knowledge on these circulating biomarkers could lead to better guidance on the appropriate therapy to deploy, in line with the concept of precision medicine. By taking advantage of exosomal release into biological fluids and their interaction with specific recipient cells, exosomes can be developed into vehicles for the delivery of drugs and other functional molecules, like miRNAs, and thus they are interesting as therapeutic agents, as already tested in a large number of studies. However, additional studies in bigger cohorts and in different types of malignancies are necessary to address the robustness and the specificity of exo-miRNA detection. Identification of easier and cheaper technologies for the detection of exosomes and microRNA is really a milestone for translation to the clinical setting. Moreover, other challenges are to find additional markers for the enrichment of CAF- or tumor cell-derived exosomes, and to discriminate miRNAs derived from the tumor microenvironment from those derived from the tumor bulk. In the therapeutics setting, exosomes could represent a clever strategy to improve the accessibility of nucleic-acid therapeutics to cancer and the cells of the tumor microenvironment. 

In conclusion, thanks to their features, exo-miRNAs can provide an innovative and useful tool for clinical oncology applications.

## Figures and Tables

**Table 1:** Oncogenic and Oncosuppressor exo-miRNAs and their roles in cancer progression. ↑ means Increase, ↓ means Decrease.

Exosomal Mir	Tumor	Effect	Ref
Oncogenic mir			
miR-10b	Breast cancer	Invasion ↑	[72]
miR-223	Pancreatic cancer	Invasion↑	[73]
miR-32-5p	Hepatocellular carcinoma	Drug resistance ↑	[74]
miR-99a-5p, miR-125b-5p	Large B-cell lymphoma	Drug resistance ↑	[75]
miR-9	Glioma	Angiogenesis ↑	[76]
miR-93-5p	Oesophageal cancer	Growth ↑	[77]
miR-21,miR-378e,miR-143	Breast Cancer	EMT and stemness ↑	[54]
Oncosuppressor MIR			
miR-192	Lung adenocarcinoma	Metastasis ↓	[78]
miR-451a	Hepatocellular carcinoma	Apoptosis ↑, Angiogenesis ↓	[79]
miR-9	Nasopharyngeal carcinoma	Angiogenesis ↓	[80]
miR-8073	Colorectal cancer	Growth ↓	[81]
miR-100	Breast cancer	Angiogenesis ↓	[82]
miR-16-5p	Mesothelioma	Growth ↓, migration ↓, invasion ↓	[83]
miR-199a	Glioma	Growth ↓, migration ↓, invasion ↓	[84]
